# Response to: Critical commentary on the association between thiazolidinedione use and dementia risk in patients with type 2 diabetes

**DOI:** 10.1111/1753-0407.13408

**Published:** 2023-05-11

**Authors:** Houyu Zhao, Lin Zhuo, Yexiang Sun, Peng Shen, Hongbo Lin, Siyan Zhan

**Affiliations:** ^1^ Department of Epidemiology and Biostatistics, School of Public Health Peking University Beijing China; ^2^ Key Laboratory of Epidemiology of Major Diseases (Peking University), Ministry of Education Beijing China; ^3^ Research Center of Clinical Epidemiology Peking University Third Hospital Beijing China; ^4^ Yinzhou District Center for Disease Control and Prevention Ningbo China; ^5^ Center for Intelligent Public Health Institute for Artificial Intelligence, Peking University Beijing China

We thank Wasif et al for their comments^1^ on our article that explored the association between thiazolidinedione (TZD) use and dementia risk in patients with type 2 diabetes mellitus (T2DM).^2^ In their letter, the authors addressed the potential confounding effects of age of diabetes onset and mental disorders, which were suggested to be associated with dementia risk in recent studies,^3^, ^4^ and thus they suggested subgroup analyses according to participants' age at T2DM onset and status of mental disorders.^1^ In our analyses we controlled age and years of T2DM at baseline^2^; thus the issue of collinearity would arise in the multivariate and inverse probability weighted model if we controlled age of T2DM onset, which was the difference between baseline age and diabetes duration. Moreover, comorbidities were adjusted using the Charlson comorbidity index, which did not contain mental disorders.^2^

In response to the authors' concerns, we conducted subgroup analyses according to their suggestions^1^ (Table 1). Age of diabetes onset was defined as the age when the patient was first diagnosed with T2DM. In addition, history of mental disorders was identified using the *International Classification of Diseases, Tenth Revision* (all codes of F10–F99 except F70–F79) according to a previous study.^4^ Consistent with previous studies, we observed higher incidence of dementia in patients of older age and with history of mental disorders.^3^, ^4^ But no significant effect modification was observed. Compared with use of alpha‐glucosidase inhibitors, TZD use was significantly associated with dementia incidence in different age groups, with a hazard ratio (HR) of 0.44 (95% confidence interval [CI], 0.21–0.91), 0.62 (95% CI, 0.39–0.98), and 0.47 (95% CI, 0.31–0.71) for T2DM patients aged <55 years, 55–65 years, and >65 years, respectively. Similarly, TZD use was consistently associated with dementia risk in terms of history of mental disorders, with an HR of 0.53 (95% CI, 0.30–0.94) and 0.50 (95% CI, 0.36–0.69) for T2DM patients with and without history of any mental disorders, respectively.

**TABLE 1:** Association between TZD use and incidence of dementia stratified by age at T2DM diagnosis and mental disorder under different outcome definitions.

Subgroup analyses	AGI users		TZD users		
	Cases/PY	Incidence (/100 000 PY)	Cases/PY	Incidence (/100 000 PY)	HR (95% CI)
Primary outcome definition^a^					
Age at T2DM diagnosis					
<55	94/129415	72.6	9/37280	24.1	0.44 (0.21–0.91)
55–65	152/93546	162.5	24/26997	88.9	0.62 (0.39–0.98)
>65	307/59597	515.1	27/12471	216.5	0.47 (0.31–0.71)
Any mental disorder					
No	415/231879	179.0	45/62197	72.4	0.50 (0.36–0.69)
Yes	138/50678	272.3	15/14551	103.1	0.53 (0.30–0.94)
Outcome definition 2^b^					
Age at T2DM diagnosis					
<55	92/129365	71.1	9/37273	24.1	0.45 (0.22–0.93)
55–65	149/93462	159.4	22/26990	81.5	0.59 (0.37–0.96)
>65	303/59385	510.2	27/12404	217.7	0.48 (0.32–0.72)
Any mental disorder					
No	407/231583	175.7	44/621146	70.8	0.50 (0.36–0.69)
Yes	137/50629	270.6	14/14521	96.4	0.52 (0.29–0.93)
Outcome definition 3^c^					
Age at T2DM diagnosis					
<55	62/129421	47.9	5/37282	13.4	0.32 (0.12–0.83)
55–65	99/93590	105.8	13/27006	48.1	0.54 (0.29–1.01)
>65	189/59652	316.8	16/12426	128.8	0.44 (0.26–0.75)
Any mental disorder					
No	263/231917	113.4	26/62178	41.8	0.46 (0.30–0.71)
Yes	87/50747	171.7	8/14536	55.0	0.39 (0.18–0.87)
Outcome definition 4^d^					
Age at T2DM diagnosis					
<55	101/129356	78.1	11/37264	29.5	0.48 (0.25–0.93)
55–65	166/93444	177.6	28/26961	103.9	0.64 (0.42–0.98)
>65	327/59388	550.6	29/12403	233.8	0.46 (0.31–0.68)
Any mental disorder					
No	445/231582	192.2	49/62129	78.9	0.50 (0.37–0.68)
Yes	149/50606	294.4	19/14500	131.0	0.56 (0.33–0.93)
Outcome definition 5^e^					
Age at T2DM diagnosis					
<55	143/129255	71.6	18/37242	48.3	0.58 (0.34–0.98)
55–65	226/93315	189.9	40/26932	148.5	0.68 (0.47–0.98)
>65	490/59122	603.4	57/12368	460.9	0.59 (0.44–0.78)
Any mental disorder					
No	648/231230	280.2	81/62063	130.5	0.57 (0.45–0.73)
Yes	211/50463	418.1	34/14477	234.8	0.73 (0.51–1.08)

Abbreviations: AGI, alpha‐glucosidase inhibitor; CI, confidence interval; HR, hazard ratio; PY, person years; TZD, thiazolidinedione; T2DM, type 2 diabetes mellitus.

^a^ An incident dementia case was defined as (a) having at least two consecutive diagnosis code or description of F00–F03 or G30, or (b) having at least one dementia diagnosis record and prescription records of antidementia drugs. Details of outcome definitions have been published previously.^2^

^b^ Outcome definition 2: Dementia defined as having at least two diagnoses of F00–F03 or G30;

^c^ Outcome definition 3: Dementia defined as having over two diagnoses of F00–F03 or G30 and prescriptions of antidementia agents after the first diagnosis;

^d^ Outcome definition 4: Dementia defined as having a consecutive diagnosis of F00–F03 or G30 or prescription of antidementia agents within 1 year of the first diagnosis of dementia;

^e^ Outcome definition 5: Dementia defined as any diagnosis of F00–F03 or G30.

We agree with the authors that we cannot fully rule out biases caused by unmeasured confounding and inaccurate information.^5^ Therefore, we conducted several sensitivity analyses using different dementia identification rules combing diagnosis and prescriptions of antidementia drugs. Results under various outcome definitions were consistent (the Table 1), suggesting that our results were robust against potential outcome misclassification.^2^ Furthermore, we did not adjust full medication history and some other potential confounders, such as dietary patterns, hence we calculate the E‐value as a sensitivity analysis. The E‐value for the observed HR of 0.51 in our primary analysis was 3.33, which was the minimum strength that an unmeasured confounder needed to be associated with both the exposure and outcome to explain away the observed association.^2^

However, our analyses should be interpreted in the context of its observational nature. Future prospective studies with more refined measurement of dementia cases and potential confounders are needed to give further insights into the potential role of TZDs in reducing dementia incidence.

## AUTHOR CONTRIBUTIONS

Houyu Zhao conceived of and designed the work. Hongbo Lin, Peng Shen, and Yexiang Sun acquired the data. Houyu Zhao analyzed the data. Houyu Zhao drafted the manuscript. Lin Zhuo and Siyan Zhan critically revised the manuscript for important intellectual content. Siyan Zhan, Hongbo Lin, and Peng Shen supervised the study. Houyu Zhao and Siyan Zhan obtained the funding. All authors were responsible for the interpretation of the data, and revised, and gave final approval of the manuscript. Siyan Zhan is the guarantor of this work and, as such, had full access to all the data in the study and takes responsibility for the integrity of the data and the accuracy of the data analysis.

## FUNDING INFORMATION

This work was supported by a grant from the China Postdoctoral Science Foundation (Grant No. 2022M710251) and a grant from the National Natural Science Foundation of China (Grant No. 81973146). The funder had no role in the study design, data collection, data analysis, data interpretation, writing of the report, or decision to submit the article for publication. The corresponding author had full access to all the data in the study and had final responsibility for the decision to submit the article for publication.

## CONFLICT OF INTEREST STATEMENT

The authors have no conflicts of interest to declare.
